# Association of CHA2DS2-VASC Score with in-Hospital Cardiovascular Adverse Events in Patients with Acute ST-Segment Elevation Myocardial Infarction

**DOI:** 10.1155/2022/3659381

**Published:** 2022-09-23

**Authors:** Caoyang Fang, Zhenfei Chen, Jing Zhang, Xiaoqin Jin, Mengsi Yang

**Affiliations:** ^1^Department of Cardiology, The Second People's Hospital of Hefei, Hefei Hospital Affiliated to Anhui Medical University, Hefei 230011, Anhui, China; ^2^Hefei Second People's Hospital Affiliated to Bengbu Medical College, Department of Cardiology, Hefei 230011, Anhui, China

## Abstract

**Background:**

Acute ST-elevation myocardial infarction (STEMI) is a common clinical critical illness, and accurate, reliable, simple, and easy-to-remember tools are needed in clinical practice to quickly identify the risk of this condition in STEMI patients. This study investigates the predictive value of the admission CHA2DS2-VASc score for in-hospital MACE in STEMI patients.

**Methods:**

A total of 210 STEMI patients who visited the Chest Pain Center of the Second People‘s Hospital of Hefei from December 2019 to December 2021 were retrospectively analyzed. They were divided into MACE and non-MACE groups. The receiver operating characteristic curve (ROC) was used to assess the predictive value of the CHA2DS2-VASc score for MACE events during hospitalization.

**Results:**

The CHA2DS2-VASc score was higher in the MACE group than in the non-MACE group (*P* < 0.05), and multivariate logistic regression analysis showed that the CHA2DS2-VASc score was an independent risk factor for MACE events during hospitalization in STEMI patients (OR = 1.391, 95%CI 1.044–1.853, *P*=0.024); ROC curve analysis showed that the area under the curve (AUC) of the CHA2DS2-VASc score was 0.744, the sensitivity was 0.64, the specificity was 0.694, and the optimal cutoff value was 3.5 in predicting the risk of MACE events during hospitalization in STEMI patients. There were no significant differences between the GRACE score (0.744 VS.0.827) and TIMI score (0.744VS.0.745) (*P* > 0.05).

**Conclusion:**

The CHA2DS2-VASc score can successfully predict the occurrence of in-hospital MACE events in STEMI patients.

## 1. Introduction

With the maturity and promotion of emergency interventional technology for acute ST-segment elevation myocardial infarction (STEMI) in clinical practices, emergency interventional therapy has become the most suitable way to treat diagnosed patients [1]. Emergency interventional therapy methods have many advantages and can improve prognosis [2], but for acute and critical diseases such as STEMI, even if patients are treated with emergency interventional methods, adverse events may still occur before, during, and after the surgical procedure, such as cardiogenic shock and sudden death [3]. Existing guidelines recommend early and continuous risk stratification for patients with acute STEMI to evaluate the prognosis and guide treatment [1]. So, it is essential to risk-stratify patients with myocardial infarction undergoing emergency interventional therapy. The TIMI score [4, 5] and GRACE score [6, 7] are the most commonly used assessment models recommended by current guidelines to predict the short-term and long-term risk of death in patients with acute STEMI. However, their calculation is complex, often requiring the use of specialized software programs, which limits their application in clinical practice [4, 6].

The CHA2DS2-VASc score is a classical scoring system constructed to assess the thromboembolic risk and guide anticoagulant therapy in patients with atrial fibrillation and considers variables such as age (≥75 years: 2 points; 65–74 years: 1 point), female gender (1 point), heart failure (1 point), hypertension (1 point), diabetes (1 point), stroke (2 points), and vascular disease (1 point), which are easy to calculate [8]. The use of the CHA2DS2-VASc score has not only been validated in a population with nonvalvular atrial fibrillation but specific parameters have also been reported as independent predictors of stroke and death in the general population as well as in patients with coronary heart disease [9]. Studies have shown that the CHA2DS2-VASc score accurately predicts adverse events after acute coronary syndrome (ACS) [10]. However, there is a research gap when it comes to the application of the CHA2DS2-VASc score and the occurrence of MACE events during hospitalization in STEMI patients. Therefore, aiming to bring novel insights regarding this correlation, this study focuses on the predictive value of the CHA2DS2-VASc score for MACE events during hospitalization in STEMI patients to create a simple, cost-effective evaluation method that can provide a valuable reference for the prognosis of STEMI patients.

## 2. Materials and Methods

### 2.1. Study Population

A total of 210 patients (162 males and 48 females, mean age 61.72 ± 14.12 years) diagnosed with acute ST-segment elevation myocardial infarction (STEMI) from December 2019 to December 2021 were retrospectively analyzed. The STEMI diagnostic criteria [11] were defined as the typical rise and fall of cardiac biomarkers, as well as at least one of the following: ① Ischemic symptoms; ② The development of pathological *Q* waves in the electrocardiogram; ③ New significant ST segment or T wave changes or new left bundle branch block; ④ Angiographic diagnosis of coronary artery disease. Exclusion criteria: ① Severe liver and kidney dysfunction; ② Infectious diseases; ③ Autoimmune diseases; ④ Blood system diseases; ⑤ Valvular diseases. This study has been approved by the Second People‘s Hospital of Hefei ethics committee (Approval No.: 2020-ke-058). All methods were performed following the Declaration of Helsinki.

### 2.2. General Data, Interventional Data, And Auxiliary Examination

Patient demographics and clinical and laboratory data were collected. First, the medical records of the patients were consulted through the hospital's electronic case system to record the general conditions and vital signs at admission, including age, gender, BMI, diabetes, hypertension, smoking, heart rate, systolic blood pressure, diastolic blood pressure, and Killip class. Then, intervention-related data were recorded according to angiography and surgical procedures. On the morning of the second day of admission, before their first meal, 5 ml of cubital venous blood was collected from each patient for blood routine and biochemical parameters. The CHA2DS2-VASc score at admission was used as a parameter to predict the occurrence of MACE events during hospitalization in STEMI patients. The CHA2DS2-VASc score was performed according to the general data of the subjects, including congestive heart failure, hypertension, diabetes, vascular disease, age between 65 and 74 years old, gender (female), smoking, family history of cardiovascular disease (with 1 point each), stroke or thrombosis, age ≥75 years (2 points). The maximum score considering all these variables was 9 points [12].

### 2.3. Definition and Grouping of MACE Events

MACE events include primary endpoint events, i.e., those with all-cause mortality. Secondary endpoint events are remyocardial infarction, reemergency revascularization, sudden cardiac arrest, heart failure, cardiogenic shock, malignant arrhythmia (including tachycardia/ventricular fibrillation, sinus arrest, high-grade or third-degree atrioventricular block), mechanical complications of myocardial infarction, stroke, and severe bleeding (hemoglobin drop ≥3 g/L). Patients with STEMI were divided into MACE group (*n* = 50) (*n* = 33, mean age 69.2 ± 13.36 years) and non-MACE group (*n* = 160) (*n* = 129, mean age 59.39 ± 13.56 years) according to the presence or absence of MACE events within 15 days after hospitalization.

### 2.4. Statistical Methods

Statistical analysis was performed using SPSS 26.0 and MedCalc 20.1.0 and plotted using GraphPad Prism9.0. A Kolmogorov-Smirnov normality test was performed for measurement data, and the normal distribution was expressed as mean ± standard deviation. An independent sample *t*-test was used for comparison between the two groups. The nonnormally distributed measurement data were expressed as median M (P25, P75). Mann-Whitney *U* test was used for comparison, and the adoption rate of enumeration data was expressed. The Chi-square test was used for comparison between the two groups. Univariate and multivariate logistic regression analysis was used to evaluate whether the CHA2DS2-VASc score could be used as an independent risk factor for MACE events in STEMI patients during hospitalization. A ROC curve was drawn to assess the predictive ability of the CHA2DS2-VASc score, GRACE score, and TIMI scores for the risk of MACE events in STEMI patients during hospitalization. The AUC of each group was compared by the Delong test [13]. All statistics were performed using two-sided tests and *P* < 0.05 were considered statistically significant.

## 3. Results

### 3.1. Comparison of Clinical Basic Data and Interventional Data between MACE Group and Non-MACE Group

There were significant differences between the MACE group and the non-MACE group in age, gender, hypertension, smoking, systolic blood pressure, diastolic blood pressure, neutrophils, hemoglobin, triglycerides, total cholesterol, LDL-C, creatinine, fasting blood glucose, LVEF, CHA2DS2-VASc score, Killip ≥ grade 2, culprit vessel as left main (LM), number of diseased vessels as single and three, whether a stent was implanted, and whether IABP was used (*P* < 0.05). However, as shown in Table 1, no significant differences were found in BMI, diabetes, heart rate, platelets, HDL-C, uric acid, and other parameters in interventional data (*P* > 0.05).

### 3.2. Analysis of Risk Factors of MACE Events in STEMI Patients

Taking the occurrence of MACE events as the endpoint, a univariate logistic regression analysis was conducted, including age, gender, hypertension, smoking, neutrophils, hemoglobin, triglycerides, total cholesterol, LDL-C, creatinine, fasting blood glucose, and CHA2DS2-VASc score. The factors with statistical significance were included in the subsequent multivariate logistic regression analysis. As shown in Table 2, the results showed that the CHA2DS2-VASc score (OR = 1.391, 95% CI 1.044–1.853, *P*=0.024) was an independent predictor of MACE events in STEMI patients during hospitalization.

### 3.3. Predictive Value of a for in-Hospital MACE Events in Patients with STEMI

**Figure 1:**
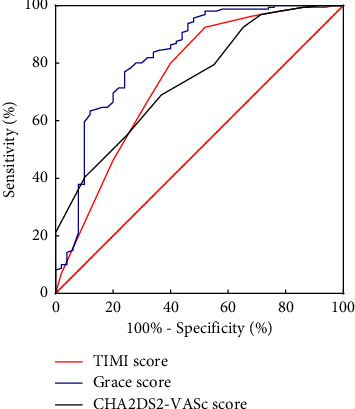
ROC curve of different scores predicting the risk of MACE events during hospitalization in STEMI patients.

According to receiver operating characteristic (ROC) curve analysis, it was identified that the area under the curve (AUC) of the CHA2DS2-VASc score was 0.744. Furthermore, some of the values found in predicting the risk of MACE events during hospitalization in STEMI patients were: (i) sensitivity = 0.64, (ii) specificity = 0.694, and (iii) optimal cutoff value = 3.5. No significant differences were found in the area under the curve (AUC) of CHA2DS2-VASc score when predicting the occurrence of MACE events during hospitalization in STEMI patients between 0.744 (95% CI: 0.67–0.819) and GRACE score (AUC: 0.827, 95% CI: 0.754–0.901, *P*=0.0809) and TIMI score (AUC: 0.745, 95% CI: 0.779–0.91, *P*=0.9862), as represented in Figure 1 (Table 3).

## 4. Discussion

Acute ST-segment elevation myocardial infarction is a common high-risk coronary atherosclerotic heart disease, which mainly occurs in elderly individuals [14, 15]. Coronary stent implantation is one of the most commonly used methods to treat this condition since it can remarkably improve the local blood flow of patients to relieve clinical symptoms. However, coronary stenting may impose some risks to the patient, and postoperative adverse events include all-cause death, remyocardial infarction, reemergency revascularization, cardiac arrest, heart failure, cardiogenic shock, malignant arrhythmias (including ventricular tachycardia), tachycardia/ventricular fibrillation, sinus arrest, high- or third-degree atrioventricular block, mechanical complications linked to myocardial infarction, stroke, and major bleeding (hemoglobin drop ≥3 g/L). Clinicians and researchers have been studying methods to ensure the efficacy of this surgical procedure while reducing the risk of postoperative adversities, with a particular focus on improving the prognosis of patients by proposing effective methods for predicting postoperative adverse events that might result from targeted interventions.

Scoring systems developed in previous studies, such as the TIMI score [4] and the GRACE score [6], have been shown to have predictive value for mortality and adverse events in patients with acute STEMI. These risk assessment models include different variables, e.g., clinical features, physical examinations, and auxiliary examinations. In clinical practice, clinicians need accurate, reliable, simple, and easy-to-remember tools to rapidly identify patients at risk for acute STEMI.

The CHA2DS2-VASc score is a classic scoring system constructed to assess the risk of thromboembolism in patients with atrial fibrillation and guide anticoagulation therapy. It includes variables such as age, female, heart failure, hypertension, diabetes, stroke, and vascular disease [16]. The variables that comprise the CHA2DS2-VASc score have been extensively studied as risk factors for death and adverse events in patients with acute STEMI [17–19]. In addition, numerous studies have shown that that CHA2DS2-VASc score can predict the prognosis of patients with various cardiovascular diseases, regardless of atrial fibrillation [20–23]. In patients diagnosed with the acute coronary syndrome, the CHA2DS2-VASc score was also associated with adverse cardiovascular events.

Rozenbaum et al. conducted a study involving 13,422 patients with acute coronary syndromes, elevated CHA2DS2-VASc score were associated with 30 day, 1 year death, and MACE [24]. Kim et al. [25] found that the CHA2DS2-VASc score could be an independent predictor of in-hospital and long-term prognosis in patients with acute myocardial infarction regardless of atrial fibrillation. Bombay et al. [18] studied the same phenomenon and found that patients with a high CHA2DS2-VASc score had higher in-hospital and long-term mortality. Peng et al. [26] found that in-hospital and long-term MACE surged with the increase of the CHA2DS2-VASc score, and the CHA2DS2-VASc score had an independent predictive value for MACE. The present study also confirms that the CHA2DS2-VASc score can be considered an independent predictor of MACE events during hospitalization in patients with acute ST-segment elevation myocardial infarction.

The results of this study showed that age, gender, history of hypertension, and smoking history in the MACE event group and the nonMACE event group were significantly different in the factors constituting the CHA2DS2-VASc score (), which is consistent with the results of previous studies (*P* < 0.05) [17–19]. Moreover, in the MACE event group, the combined left main disease and three-vessel disease were higher than those in the nonMACE event group, and the single-vessel disease was lower, so the differences were considered statistically significant. These results confirm that patients in the MACE event group were more prone to cardiovascular adverse events after acute ST-segment elevation myocardial infarction.

Furthermore, compared with the nonMACE event group, the CHA2DS2-VASc score of the MACE event group was higher, and the difference was statistically significant. Results from the multivariate logistic regression analysis showed that neutrophil count, creatinine, and CHA2DS2-VASc score were independent risk factors for MACE events during hospitalization in STEMI patients. This result is associated with the fact that within 24 hours of admission, the CHA2DS2-VASc score was considered an independent risk factor for MACE events during hospitalization in STEMI patients. Therefore, it is further speculated that the CHA2DS2-VASc score may also play a role in predicting the occurrence of MACE events during hospitalization in STEMI patients. Therefore, the ROC curve analysis found that the area under the curve (AUC) of the CHA2DS2-VASc score in predicting the occurrence of MACE events was 0.744 (95% CI 0.67–0.819, *P* < 0.001). The predictive value of the risk of developing a MACE event was comparable to the GRACE score and the TIMI score, which indicates that the CHA2DS2-VASc score had a good predictive value for the occurrence of MACE events. Compared with the GRACE score and TIMI score, the CHA2DS2-VASc score has many advantages in evaluating the occurrence of MACE events during hospitalization in STEMI patients. The variables included in the CHA2DS2-VASc score are all clinical data that can be obtained for the first time when the patients are admitted to the hospital. The calculation is simple and suitable for rapid bedside assessment [8].

The results shared in this study prove that the CHA2DS2-VASc score can provide an early basis for the occurrence of MACE events during hospitalization in STEMI patients before obtaining data such as ECG, laboratory tests, and coronary angiography. Since the 2010 European Society of Cardiology guidelines for the treatment of atrial fibrillation recommended it for thromboembolic risk assessment in patients with atrial fibrillation, the CHA2DS2-VASc score has been widely used in clinical practice and is well-known by the majority of medical workers, which is conducive to its promotion and application in the risk assessment of whether MACE events occur during hospitalization in STEMI patients.

It is equally important to acknowledge the limitations of this research. First, this is a single-center retrospective study with a limited sample size, which may have led to some biased conclusions. Second, the research design only explored the immediate predictive value of the CHA2DS2-VASc score for the occurrence of MACE events during hospitalization in STEMI patients without considering changes that could happen during a long-term followup period. In the future, multi-center, large-scale, and prospective trials are still needed for further verification.

## 5. Conclusion

In conclusion, the results of this study generally suggest that the CHA2DS2-VASc score is an independent risk factor for in-hospital MACE events during hospitalization in STEMI patients. The CHA2DS2-VASc score is comparable to the GRACE score and TIMI score in predicting the risk of MACE events under these conditions, and the related data are easy to obtain, simple to calculate, and suitable for rapid bedside assessment. It can also provide a valuable reference for the prognosis of STEMI patients.

## Figures and Tables

**Table 1:** Comparison of data between the MACE group and the nonMACE group.

Variables	MACE group	Non-MACE group	*t*/*χ*^*2*^/*z*-value	*P* value
Clinical data				
Age (years)^a^	69.2 ± 13.36	59.39 ± 13.56	4.481	<0.001^*∗*^
Gender (male, *n*%)	33 (66)	129 (80.6)	4.621	0.032^*∗*^
BMI (kg/m^2^)^a^	24.25 ± 3.94	24.73 ± 3.39	0.672	0.502
Diabetes (*n*%)	21 (15.8)	37 (23.1)	3.17	0.075
Hypertension (*n*%)	36 (72)	83 (51.9)	6.283	0.012^*∗*^
Smoking (*n*%)	20 (40)	96 (60)	6.163	0.013^*∗*^
Heart rate (beats/min)^a^	84.7 ± 22.51	77.92 ± 15.65	1.986	0.051
Systolic blood pressure (mmHg)^a^	115.82 ± 28.32	126.13 ± 22.84	2.625	0.009^*∗*^
Diastolic blood pressure (mmHg)^a^	68.42 ± 15.28	77 ± 16.69	3.236	0.001^*∗*^
Killip≥2 (*n*%)	33 (66)	33 (20.6)	36.395	<0.001^*∗*^
LVEF^a^	51.58 ± 10.31	58.55 ± 7.22	4.45	<0.001^*∗*^
CHA2DS2-VASc group^b^	4 (2.75,7)	2 (1,4)	5.264	<0.001^*∗*^

Laboratory data				
Neutrophils(×10^9^/l)^b^	8.94 (6.28,12.29)	7.65 (5.52,9.82)	2.65	0.008^*∗*^
Hemoglobin (g/L)^a^	127.32 ± 18.04	137.81 ± 19.55	3.372	0.001^*∗*^
Platelets (×10^9^/l)^b^	189 (153,240)	201.5 (150,242)	0.736	0.462
Triglycerides (mmol/L)^b^	1.22 (0.75,1.68)	1.51 (1.06,2.14)	2.402	0.016^*∗*^
Total cholesterol (mmol/L)^b^	3.98 (3.49,4.7)	4.61 (3.91,5.31)	3.114	0.002^*∗*^
LDL-c (mmol/L)^b^	2.39 (1.86,3.26)	2.94 (2.35,3.5)	2.965	0.003^*∗*^
HDL-c (mmol/L)^b^	1.03 (0.86,1.31)	1.06 (0.92,1.22)	0.871	0.384
Creatinine (umol/l)^b^	83.05 (58.08,113.23)	66.9 (56.18,75.98)	3.532	<0.001^*∗*^
Uric acid (umol/L)^b^	380.3 (275.13,446.15)	339.3 (282.9,418.95)	1.249	0.212
Fasting blood glucose (mmol/L)^b^	8.51 (6.33,12.63)	6.14 (5.34,8.09)	3.814	<0.001^*∗*^

Interventional data				
Criminal vessel				
LM, (*n*%)	3 (6)	0 (0)	9.739	0.002^*∗*^
LAD, (*n*%)	24 (48)	91 (56.9)	1.211	0.271
LCX, (*n*%)	9 (18)	17 (10.6)	1.91	0.167
RCA, (*n*%)	22 (44)	55 (34.4)	1.52	0.218
Number of diseased vessels				
One, (*n*%)	7 (14)	57 (35.6)	8.408	0.004^*∗*^
Two, (*n*%)	17 (34)	55 (34.4)	0.002	0.961
Three, (*n*%)	26 (52)	48 (30)	8.079	0.004^*∗*^
Stent implantation,(*n*%)	41 (82)	148 (92.5)	4.667	0.031^*∗*^
Number of stents implanted,(*n*%)	1 (1,2)	1 (1,2)	0.074	0.941
Stent length^b^	23 (18,29)	23 (18,29)	0.779	0.436
Stent diameter^b^	2.75 (2.75,3)	3 (2.75,3)	0.44	0.66
Tirofiban, (*n*%)	21 (42)	53 (33.1)	1.315	0.252
Thrombus aspiration, (*n*%)	13 (26)	25 (15.6)	2.767	0.096
IABP, (*n*%)	12 (24)	4 (2.5)	25.019	<0.001^*∗*^

[Mean ± standard deviation, M (P_25_, P_75_), number of cases and percentage (*n*%)]. BMI : Body mass index; LDL-C: low-density lipoprotein cholesterol C; HDL-C: high-density lipoprotein cholesterol C; LVEF : Left ventricular ejection fraction; LM : Left main coronary artery; LAD : Left anterior descending artery; LCX : Left circumflex; RAC : Right coronary artery; IABP : Aortic balloon counterpulsation;^a^:Normally distributed data are expressed as mean ± standard deviation;^b^: Nonnormally distributed data are expressed as median M (P_25_, P_75_); ^*∗*^:*P* < 0.05.1 mmHg = 0.133 kPa.

**Table 2:** Logistic regression analysis of risk factors in the MACE group.

	Univariate logistic regression analysis				Multivariate logistic regression analysis			
	*β*	OR	95%CI	*P* value	*β*	OR	95% CI	*P* value
Age	0.054	1.056	(1.029,1.083)	<0.001	0.003	1.003	(0.957,1.052)	0.888
Gender	0.763	2.144	(1.06,4.336)	0.034	0.168	1.183	(0.359,3.904)	0.782
Hypertension	0.869	0.419	(0.21,0.836)	0.014	0.408	1.504	(0.558,4.051)	0.42
Somking	0.811	2.25	(1.177,4.302)	0.014	0.281	1.324	(0.487,3.598)	0.582
CHA2DS2-VASc group	0.42	1.522	(1.301,1.782)	<0.001	0.33	1.391	(1.044,1.853)	0.024^*∗*^
Neutrophils	0.144	1.154	(1.065,1.252)	0.001	0.177	1.194	(1.063,1.342)	0.003^*∗*^
Hemoglobin	0.027	0.973	(0.957,0.989)	0.001	0.024	0.976	(0.952,1.001)	0.059
Triglycerides	0.123	0.884	(0.655,1.194)	0.423	—	—	—	—
Total cholesterol	0.46	0.632	(0.453,0.881)	0.007	0.177	0.837	(0.334,2.099)	0.705
LDL-C	0.564	0.569	(0.379,0.855)	0.007	0.026	1.027	(0.328,3.211)	0.964
Creatinine	0.025	1.025	(1.013,1.037)	<0.001	0.02	1.02	(1.004,1.036)	0.013^*∗*^
Fasting blood glucose	0.113	1.119	(1.042,1.202)	0.002	0.087	1.09	(0.998,1.191)	0.054

LDL-C: low-density lipoprotein cholesterol C; ^*∗*^:*P* < 0.05.1.

**Table 3:** Prediction of MACE events by CHA2DS2-VASc score, Grace score, and TIMI score.

	Cut-off	Standard error	AUC (95% CI)	*P*-value	Sensitivity (%)	Specificity (%)	Youden Index
TIMI score	6.5	0.044	0.745 (0.779,0.91)	<0.001	0.48	0.925	0.405
Grace score	135	0.037	0.827 (0.754,0.901)	<0.001	0.76	0.769	0.529
CHA2DS2-VASc score	3.5	0.038	0.744 (0.67,0.819)	<0.001	0.64	0.694	0.334

## Data Availability

The datasets used and/or analyzed during the current study are available from the corresponding author on reasonable request.

## References

[1] Ibanez B., James S., Agewall S. (2018). 2017 ESC guidelines for the management of acute myocardial infarction in patients presenting with ST-segment elevation: the Task Force for the management of acute myocardial infarction in patients presenting with ST-segment elevation of the european society of cardiology (ESC). *European Heart Journal*.

[2] Brogan R. A., Alabas O., Almudarra S. (2019). Relative survival and excess mortality following primary percutaneous coronary intervention for ST-elevation myocardial infarction. *European heart journal Acute cardiovascular care*.

[3] Khan K. N., Khan M. H., Rahman R., Rashid M. A., Haque S. Z., Zakia Z. (2017). Primary angioplasty for the treatment of acute ST elevated myocardial infarction: single centre experience. *Mymensingh Medical Journal: MMJ*.

[4] Morrow D. A., Antman E. M., Charlesworth A. (2000). TIMI risk score for ST-elevation myocardial infarction: a convenient, bedside, clinical score for risk assessment at presentation: an intravenous nPA for treatment of infarcting myocardium early II trial substudy. *Circulation*.

[5] Karabağ Y., Çağdaş M., Rencuzogullari I. (2018). Comparison of SYNTAX score II efficacy with SYNTAX score and TIMI risk score for predicting in-hospital and long-term mortality in patients with ST segment elevation myocardial infarction. *The International Journal of Cardiovascular Imaging*.

[6] Granger C. B., Goldberg R. J., Dabbous O., Pieper K. S., Eagle K. A., Cannon C. P. (2003). Predictors of hospital mortality in the global registry of acute coronary events. *Archives of Internal Medicine*.

[7] Chen Y. H., Huang S. S., Lin S. J. (2018). TIMI and GRACE risk scores predict both short-term and long-term outcomes in Chinese patients with acute myocardial infarction. *Acta Cardiologica Sinica*.

[8] Wang B. Y., Lin F. Y., Ku M. S., Wang Y. H., Lee K. Y., Ho S. W. (2021). CHA2DS2-VASc score for major adverse cardiovascular events stratification in patients with pneumonia with and without atrial fibrillation. *Journal of Clinical Medicine*.

[9] Wu Y., Wang G., Dong L. (2021). Assessment of the CHA(2)DS(2)-VASc score for the prediction of death in elderly patients with coronary artery disease and atrial fibrillation. *Frontiers in cardiovascular medicine*.

[10] Borovac J. A., Kwok C. S., Mohamed M. O. (2021). The predictive value of CHA(2)ds(2)-VASc score on in-hospital death and adverse periprocedural events among patients with the acute coronary syndrome and atrial fibrillation who undergo percutaneous coronary intervention: a 10-year national inpatient sample (NIS) analysis. *Cardiovascular Revascularization Medicine*.

[11] Jaffe A. S., Alpert J. S., Jaffe A. S., Simoons M. L., Chaitman B. R., White H. D. (2013). Third universal definition of myocardial infarction. *Clinical Biochemistry*.

[12] Camm A. J., Lip G. Y. H., De Caterina R. (2012). 2012 focused update of the ESC guidelines for the management of atrial fibrillation: an update of the 2010 ESC Guidelines for the management of atrial fibrillation. developed with the special contribution of the european heart rhythm association. *European Heart Journal*.

[13] DeLong E. R., DeLong D. M., Clarke-Pearson D. L. (1988). Comparing the areas under two or more correlated receiver operating characteristic curves: a nonparametric approach. *Biometrics*.

[14] Badimon L., Bugiardini R., Cubedo J. (2016). Pathophysiology of acute coronary syndromes in the elderly. *International Journal of Cardiology*.

[15] Nammas W., de Belder A., Niemelä M. (2017). Long-term clinical outcome of elderly patients with acute coronary syndrome treated with early percutaneous coronary intervention: insights from the BASE ACS randomized controlled trial. *European Journal of Internal Medicine*.

[16] Lip G. Y., Nieuwlaat R., Pisters R., Lane D. A., Crijns H. J. (2010). Refining clinical risk stratification for predicting stroke and thromboembolism in atrial fibrillation using a novel risk factor-based approach: the euro heart survey on atrial fibrillation. *Chest*.

[17] Ashoori A., Pourhosseini H., Ghodsi S. (2019). CHA2DS2-VASc score as an independent predictor of suboptimal reperfusion and short-term mortality after primary PCI in patients with acute ST segment elevation myocardial infarction. *Medicina (Kaunas, Lithuania)*.

[18] Bozbay M., Uyarel H., Cicek G. (2017). CHA (2) DS (2)-VASc score predicts in-hospital and long-term clinical outcomes in patients with ST-segment elevation myocardial infarction who were undergoing primary percutaneous coronary intervention. *Clinical and Applied Thrombosis*.

[19] Li X., Zeng Z., Yang X., Wang H. (2021). Predictive value of CHADS (2) and CHA (2) DS (2)-VASc scores for coronary artery lesions and in-hospital prognosis of patients with acute ST-segment elevation myocardial infarction. *BMC Cardiovascular Disorders*.

[20] Reitan C., Platonov P. G., Borgquist R. (2021). The cha2ds2-VASc score and its association with long-term outcome in a cardiac resynchronization therapy population. *Cardiology*.

[21] Kuczaj A., Nowalany-Kozielska E., Skrzypek M. (2019). Relationship between the CHA2DS2-VASc score and atrial fibrillation in patients hospitalised due to heart failure. *Kardiologia Polska*.

[22] Berkovitch A., Mazin I., Younis A. (2019). CHA2DS2-VASc score performance to predict stroke after acute decompensated heart failure with and without reduced ejection fraction. *EP Europace*.

[23] Zhu W., Wu Y., Zhou Y. (2020). CHA2DS2-VASc and ATRIA scores and clinical outcomes in patients with heart failure with preserved ejection fraction. *Cardiovascular Drugs and Therapy*.

[24] Rozenbaum Z., Elis A., Shuvy M. (2016). CHA2DS2-VASc score and clinical outcomes of patients with acute coronary syndrome. *European Journal of Internal Medicine*.

[25] Kim K. H., Kim W., Hwang S. H. (2015). The CHA2DS2VASc score can be used to stratify the prognosis of acute myocardial infarction patients irrespective of presence of atrial fibrillation. *Journal of Cardiology*.

[26] Peng H., Sun Z., Chen H. (2019). Usefulness of the CHA (2) DS (2)-VASc score to predict adverse outcomes in acute coronary syndrome patients without atrial fibrillation undergoing percutaneous coronary intervention. *The American Journal of Cardiology*.

[27] Fang C., Chen Z., Zhang J. (2022). Predictive value of serum YKL-40 for major adverse cardiovascular events within 30 days after PCI for acute ST-segment elevation myocardial infarction. *Cardiology research and Practice*.

[28] Chen Z., Zhang J., Feng J., Zhou G., Jin X., Pan J. (2021). Higher serum level of cystatin C: an additional risk factor of CAD. *Medicine*.

